# Asynchronous life cycles contribute to reproductive isolation between two Alpine butterflies

**DOI:** 10.1093/evlett/qrad046

**Published:** 2023-10-07

**Authors:** Selim Bouaouina, Yannick Chittaro, Yvonne Willi, Kay Lucek

**Affiliations:** Department for Environmental Sciences, University of Basel, Basel, Switzerland; info fauna, Neuchâtel, Switzerland; Department for Environmental Sciences, University of Basel, Basel, Switzerland; Department for Environmental Sciences, University of Basel, Basel, Switzerland; Biodiversity Genomics Laboratory, Institute of Biology, University of Neuchâtel, Neuchâtel, Switzerland

**Keywords:** allochrony, biodiversity, co-existence, *Erebia*, Lepidoptera, speciation

## Abstract

Geographic isolation often leads to the emergence of distinct genetic lineages that are at least partially reproductively isolated. Zones of secondary contact between such lineages are natural experiments that allow investigation of how reproductive isolation evolves and co-existence is maintained. While temporal isolation through allochrony has been suggested to promote reproductive isolation in sympatry, its potential for isolation upon secondary contact is far less understood. Sampling two contact zones of a pair of mainly allopatric Alpine butterflies over several years and taking advantage of museum samples, we show that the contact zones have remained geographically stable over several decades. Furthermore, they seem to be maintained by the asynchronous life cycles of the two butterflies, with one reaching adulthood primarily in even and the other primarily in odd years. Genomic inferences document that allochrony is leaky and that gene flow from allopatric sites scales with the degree of geographic isolation. Overall, we show that allochrony has the potential to contribute to the maintenance of secondary contact zones of lineages that diverged in allopatry.

## Introduction

The evolution of new species by geographic isolation is among the commonest modes of speciation (Coyne et al., 2004). While such allopatric speciation may reach completion in geographic separation, diverging lineages often remain at least partially interfertile over long periods of time. When they come into secondary contact, the progression (or the collapse) of speciation very much depends on the presence and strengths of barriers to gene flow and thus on the level of reproductive isolation between them (Coyne et al., 2004; Kulmuni et al., 2020). Also, additional reproductive barriers may evolve or existing ones may be reinforced to reduce costly hybridization events upon secondary contact (Servedio & Noor, 2003). Barriers that maintain lineages in secondary contact commonly involve ecological differentiation, or differentiation in morphology, physiology, or behavior of mating (Johannesson et al., 2020; Weber & Strauss, 2016). Here, we study the potential for another type of isolating mechanism, the asynchrony in the life cycle of diverging lineages, called allochrony.

Temporal isolation through allochrony has been suggested to promote speciation in sympatry (Rosser et al., 2022). Empirical examples of sympatric speciation associated with allochrony include *Rhagoletis* fruit flies that differ in seasonal emergence and are specialized either on wild or cultivated fruits (Inskeep et al., 2022; Rosser et al., 2022), or *Coregonus* whitefish that spawn in summer or winter (Hudson et al., 2011). Allochrony may also involve temporal isolation across many years, as in North American periodical cicadas (*Magicicada* spp.), which emerge after 13 or 17 years and co-emerge only every 221 years (Simon et al., 2000). However, while temporal isolation between closely related species has been frequently documented, comparatively few examples of allochronic speciation are known reviewed in Taylor & Friesen (2017). Similar to sympatric speciation, allochrony has been suggested to provide the potential to promote co-existence or even advance speciation between diverging lineages in zones of secondary contact (Taylor & Friesen, 2017). Allochronic isolation through different migration patterns has, for example, been suggested between closely related species or lineages of birds (Green et al., 2022; Tang et al., 2022). Speciation in these systems is often advanced and gene flow is strongly limited or absent. Here, we describe a case in the genus *Erebia*, one of the most diverse Palearctic butterfly genera (Peña et al., 2015), in which gene flow is still possible.

**Figure 1.**
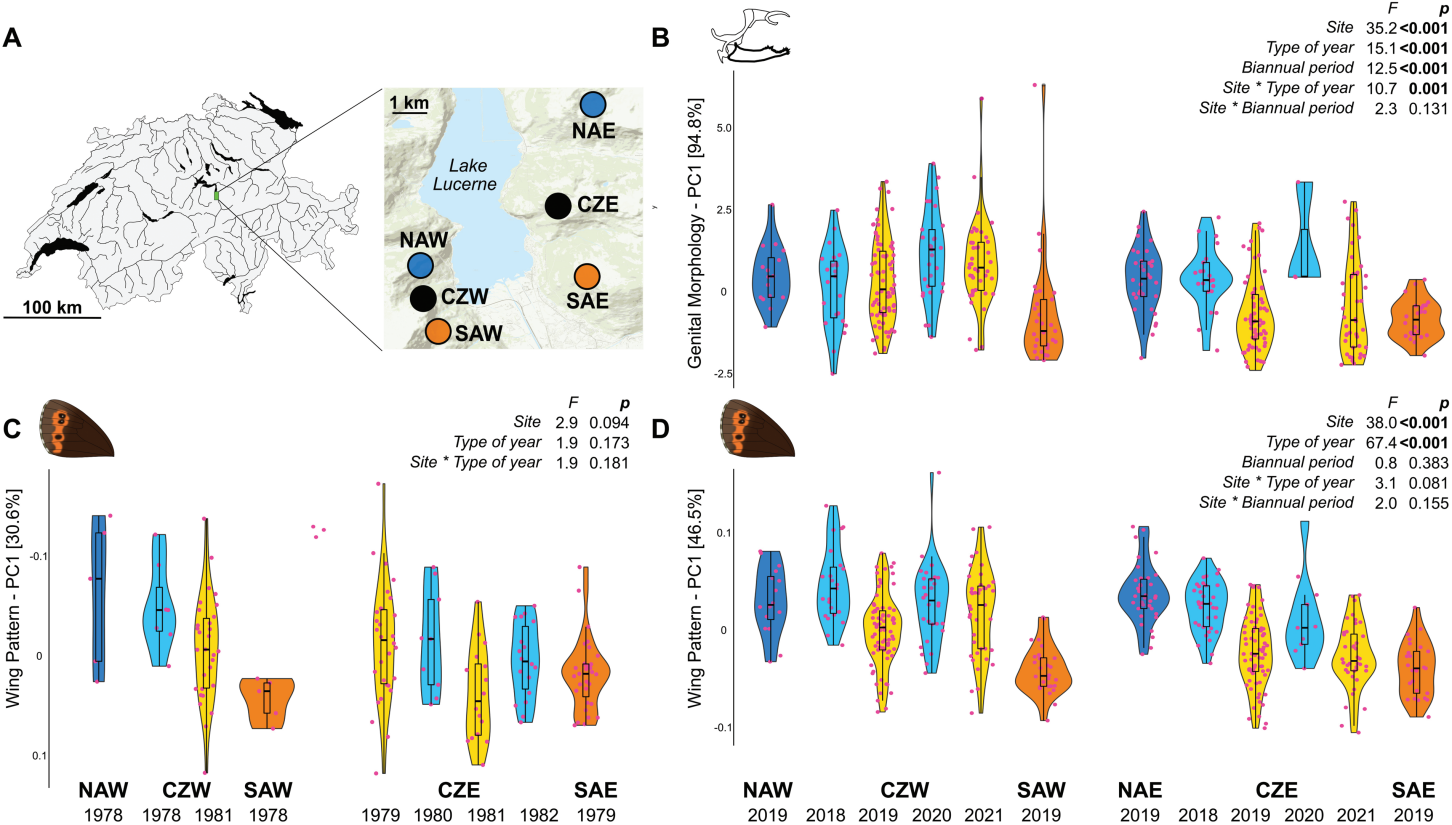
Phenotypic changes through time. (A) Map of Switzerland with sampling sites indicated: NAW/NAE = northern allopatric *E. euryale isarica* west/east; CZW/CZE = contact zone west/east of Lake Lucerne (in bright blue); SAW/SAE = southern allopatric *E. euryale adyte* west/east. (B) Violin plots for the leading principal component (PC1) on genital morphology separately for sampling site and year. (C) Violin plots for PC1 on wing pattern separately for sampling site and year for museum samples. (D) Violin plots for PC1 on wing pattern separately for sampling site and year for contemporary samples. Dots indicate individual PC scores. Dark colors indicate allopatric populations (blue = *isarica*, orange = *adyte*), and light colors alternating years of sampling at the contact zones. For B-D the respective ANOVA statistics are indicated, using *Site* (contact zone), *Type of year* (even or odd), and *Biannual period* (2018/2019 and 2020/2021) as factors (see main text for details). Because of the uneven sampling, only *Type of year* and *Site*, was included in the analysis of historic samples.

Closely related *Erebia* species and lineages often diverged from one another in distinct glacial refugia and established narrow zones of secondary contact following their postglacial range expansions (Lucek et al., 2020; Schmitt & Müller, 2007; Sonderegger, 2005). *Erebia euryale* is a common species that is widespread across Europe, occurring in light forests and meadows in the subalpine zone from Spain to Russia, without any apparent habitat-dependent differentiation between distinct glacial lineages (Cupedo, 2010, 2014; Schmitt & Haubrich, 2008; Sonderegger, 2005). Also in the Alps, closely related *E. euryale* lineages are associated with distinct glacial refugia in unglaciated foothills and peripheric nunataks surrounding the Alps (Cupedo & Doorenweerd, 2022). Like other *Erebia* species, *E. euryale* is thought to take 2 years to develop, often resulting in variable abundance of flying adults between alternating years (Kleckova et al., 2015; Sonderegger, 2005). The biennial occurrence can vary regionally, but neither the underlying biological drivers nor the potential biogeographic patterns of bienniality are understood (Kleckova et al., 2015; Sonderegger, 2005). In the current study, we focus on two of these mainly allopatric lineages from the Alps, one with a more northern and the other with a more southern distribution, *E. euryale isarica* and *E. euryale adyte* (further referred to as *isarica* and *adyte*), respectively. We studied two of their known contact zones in Switzerland that are about 8 km apart and separated by Lake Lucerne (Figure 1A). These contact zones were initially described in the 1980s as being a few hundred meters wide and with only a small number of intermediate phenotypes (Rezbanyai-Reser, 1991; Sonderegger, 2005). Similar to other *Erebia* that form secondary contact zones (Augustijnen et al., 2022; Sonderegger, 2005), *isarica* and *adyte* differ phenotypically in their wing patterns and male genital morphology, which are also traits that could be linked to prezygotic isolation through sexual selection and lock–key mechanisms, respectively. In addition, the presence of the endosymbiotic bacterium *Wolbachia* has been suggested to play a role in maintaining stable and narrow secondary contact zones in *Erebia* (Augustijnen et al., 2022; Lucek et al., 2020). However, this is unlikely the case for the studied *E. euryale* contact zones because the prevalence of *Wolbachia* is comparatively low and both lineages share the same *Wolbachia* strain (Lucek et al., 2021).

By sampling the two contact zones of *E. isarica* and *adyte* over 4 consecutive years, a the first goal was to describe patterns of phenology within and between the two lineages across time and space using morphology. Historic patterns of phenology in the contact zones were further analyzed based on museum specimens. The second goal was to assess the impact of recurrent gene flow on genetic differentiation during secondary contact, which was achieved by genomic analysis of collected samples. We predicted that gene flow was higher in the western compared to the eastern contact zone because of the lower degree of geographic isolation by topography, both among allopatric sites of the two lineages and with the contact zone (Figure 1A). A key finding was an alternating phenotypic and genetic shift between sampling years in the contact zones, consistent with the alternating presence of *isarica* and *adyte* at different frequencies, which we discuss in the context of allochrony during secondary contact.

## Results

We collected between 32 and 101 *E. euryale* specimens in each of 4 consecutive years (2018–2021) in the two contact zones in central Switzerland as well as from nearby, putatively allopatric locations (Figure 1A; Supplementary Table S1). To track phenotypic shifts among sampling years, we quantified genital morphology and wing pattern and performed principal component (PC) analyses across all contemporary individuals. The leading PC axes accounted for 94.8% and 46.5% of the total variation for genital morphology and wing pattern, respectively. For genital morphology, *contact zone* (*F*_1,295_ = 35.2, *p* < .001), *type of year* (even or odd; *F*_1,295_ = 15.1, *p* < .001), and *biannual period* (2018/2019 and 2020/2021; *F*_1,295_ = 12.5, *p* < .001) were significantly different. Here, the interaction between *type of year* and *site* (*F*_1,295_ = 10.9, *p* = .001) but not the one between *biannual period* and *site* (*F*_1,295_ = 2.3, *p* = .131) was significant, highlighting that phenotypic shifts among sampling years were more pronounced in the eastern contact zone (Supplementary Figure S1A). For wing pattern, *site* (*F*_1,315_ = 38.0, *p* < .001) and *type of year* (*F*_1,315_ = 67.4, *p* < .001) but not *biannual period* (*F*_1,315_ = 0.7, *p* = .383) were significantly different with no significant interaction (*type of year* × *site*: *F*_1,315_ = 3.1, *p* = .081; *biannual period* × *site*: *F*_1,315_ = 2.0, *p* = .155). This suggests that for wing patterns annual phenotypic shifts follow a similar trajectory among contact zones (Supplementary Figure S1B). Focusing on the four subsequent PC axes that accounted for more than 5% of the total variation in wing pattern, *type of year* was significant in three cases, and the interaction with contact zone twice (PC axes 4 and 5; Supplementary Figure S2). Overall, more individuals collected in the contact zones during even years resembled allopatric *isarica*, while more individuals from odd years resembled allopatric *adyte* (Figure 1), consistent with allochronic life cycles.

We addressed the longer-term temporal stability of allochrony in the contact zones by analyzing historic samples. We photographed the museum specimens that had been used to initially describe the contact zones, but, although collected over several years, between 1978 and 1982, had not been analyzed in regard to sampling year yet (Rezbanyai-Reser, 1991). Although we found phenotypic shifts, *i.e*., from more *isarica* like phenotypes in even to more *adyte* like phenotype in odd years for wing pattern, these were not significant along PC1 (*type of year*: *F*_1,106_ = 1.9, *p* = .173; Figure 1C) with no significant difference between contact zones (*F*_1,106_ = 2.9, *p* = .094). *Type of year* differed though for PC2 (11.1%) and PC5 (6.5%), while contact zone was never significant (Supplementary Figure S3). These results suggest that the pattern of allochrony was already present at the contact zones more than four decades ago, consistent with the previous recorded presence of both *adyte* and *isarica* (Rezbanyai-Reser, 1991).

**Figure 2.**
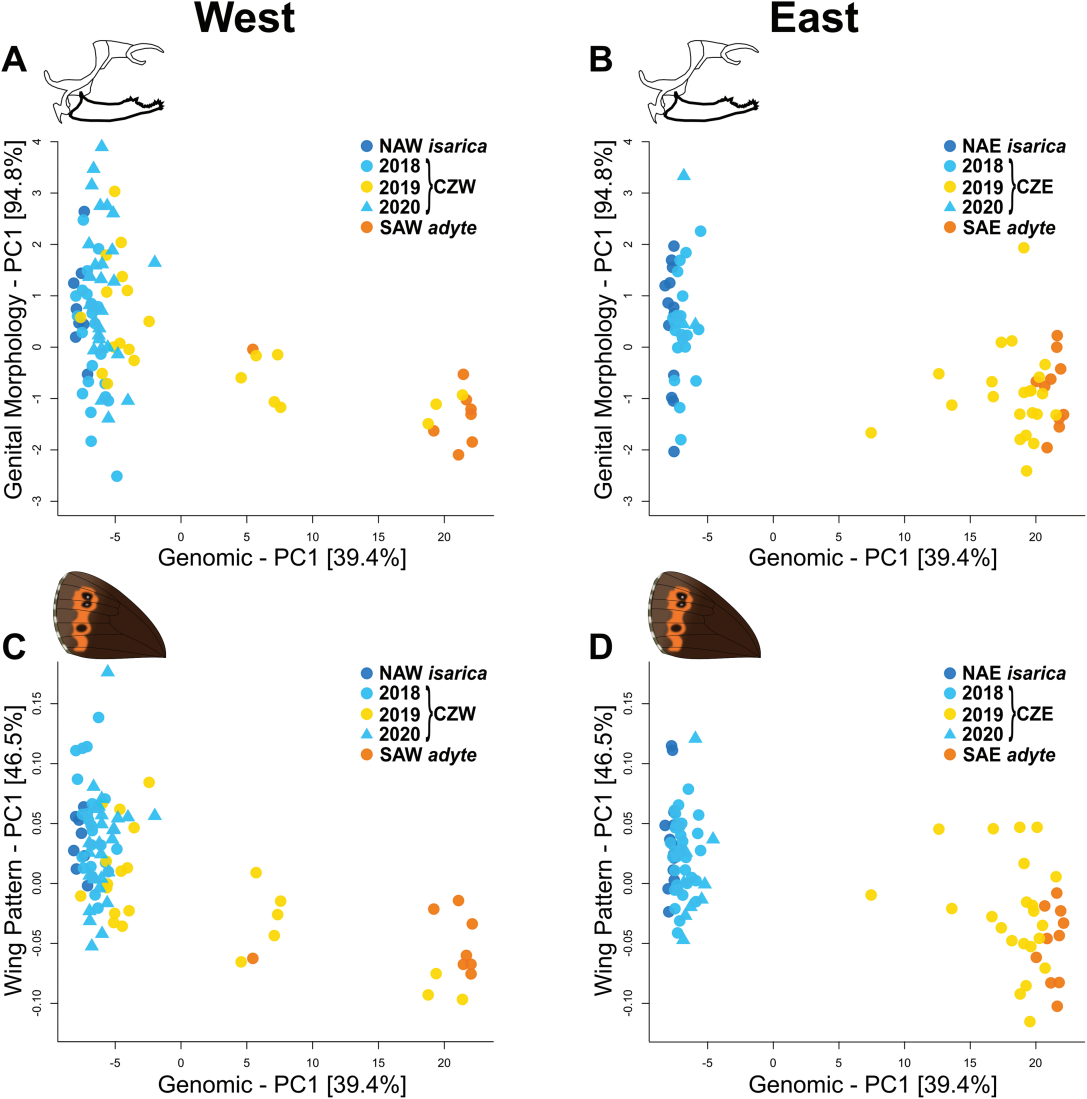
Relationship between genotypes and phenotypes. Scores of the leading principal components (PC1) of the genomic data against those of (A) genital morphology of the western contact zone, (B) genital morphology of the eastern contact zone, (C) wing pattern of the western contact zone, and (D) wing pattern of the eastern contact zone. Colors and shapes depict different sampling sites and years for each site. Dark colors indicate allopatric populations (blue = *isarica*, orange = *adyte*), light colors alternating years at the contact zones. Abbreviations: NAW/NAE = northern allopatric *E. euryale isarica* west/east; CZW/CZE = contact zone west/east, SAW/SAE = southern allopatric *E. euryale adyte* west/east.

To test to which degree these phenotypic changes are also associated with genetic shifts, we genotyped 180 contemporary individuals from the two contact zones and their respective allopatric sites using restriction-site-associated DNA (RAD) sequencing. The leading axis of the genomic PCA across all genotyped individuals accounted for 39.4% of the total variation and separated *isarica* and *adyte* (Figure 2). Its scores were significantly correlated with the scores of the leading axes of the phenotypic PCAs, for both genital morphology (west: *Pearson r* = 0.504, *t*_1,87_ = 5.4, *p* < .001; east: *r* = 0.560, *t*_1,66_ = 5.5, *p* < .001) and wing pattern (west: *r* = 0.617, *t*_1,87_ = 7.3, *p* < .001; east: *r* = 0.600, *t*_1,85_ = 6.9, *p* < .001). However, for both genital morphology and wing pattern, the scores of the phenotypic PCA overlapped between *isarica* and *adyte*, with admixed individuals having intermediate PC scores which overlapped with those of unadmixed individuals (Figure 2). This overlap highlights that wing pattern and genital morphology likely do not act as strong prezygotic barriers and that gene flow leads to intermediate phenotypes that fall within the natural spectrum of phenotypic diversity.

**Figure 3.**
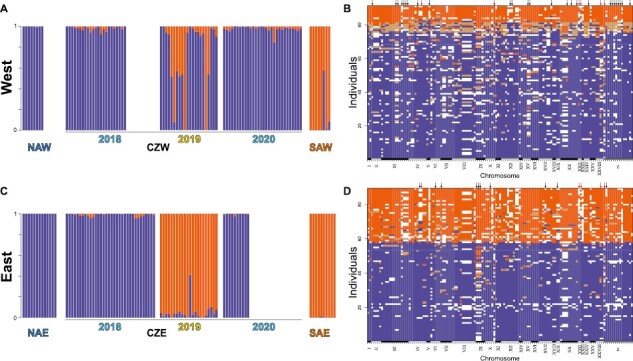
The genomic outcome of secondary contact. Individual-based assignments by ADMIXTURE analysis assuming two genetic clusters (*K* = 2) for (A) the western and (C) eastern contact zone, respectively. Fixed SNPs for allopatric *isarica* (blue) and *adyte* (orange) with respective heterozygous sites (brown) across the genome for all individuals from (B) the western and (D) eastern contact zones. White squares in B and D indicate missing data. Arrows above the allele tables indicate SNPs with significantly lower or higher introgression than expected by *introgress*. Red arrows indicate outliers that were detected in both contact zones. Abbreviations: NAW/NAE = northern allopatric *E. euryale isarica* west/east; CZW/CZE = contact zone west/east, SAW/SAE = southern allopatric *E. euryale adyte* west/east.

**Figure 4.**
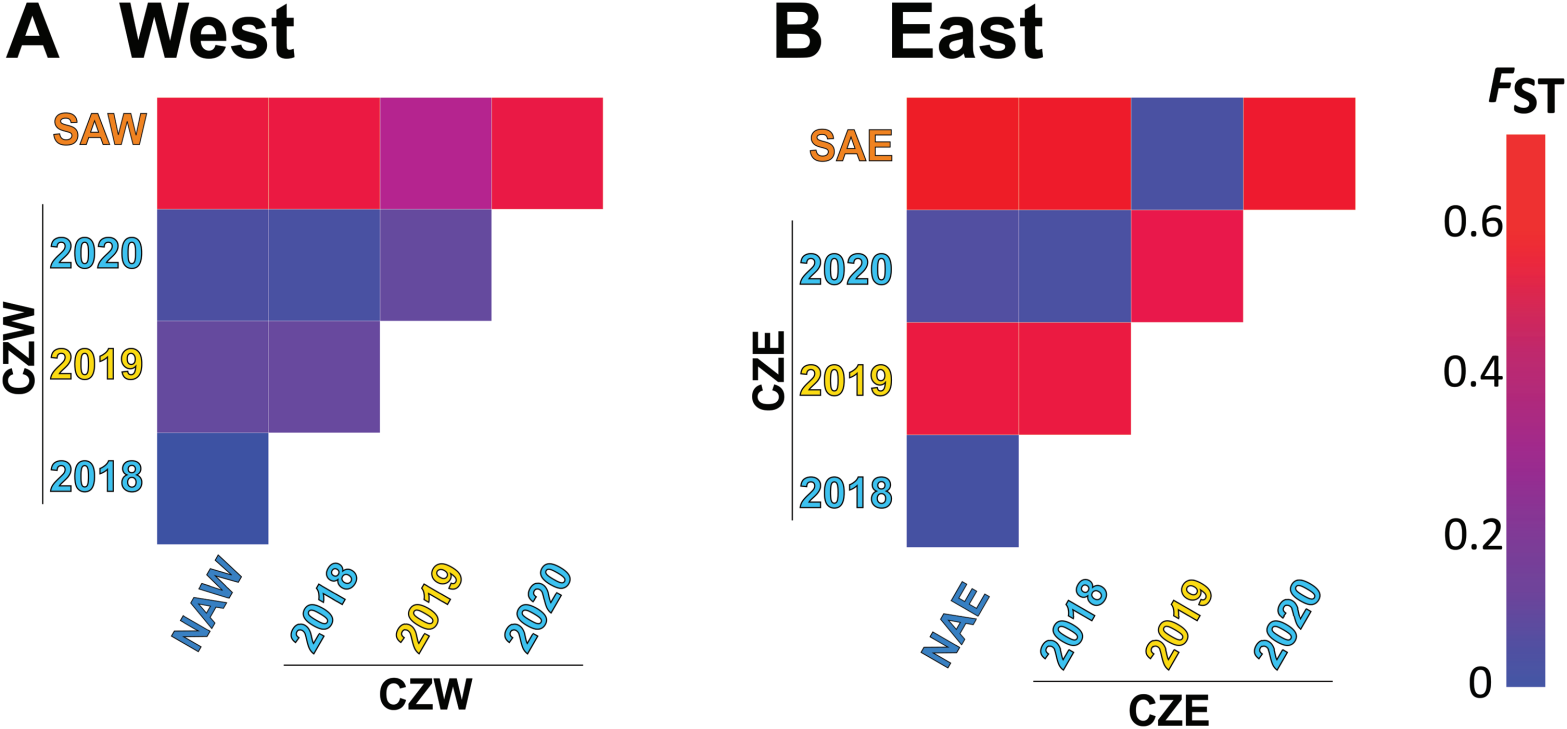
Heatmaps depicting the level of genetic differentiation among sampling sites and years for the (A) western and (B) eastern contact zones. Abbreviations: NAW/NAE = northern allopatric *E. euryale isarica* west/east; CZW/CZE = contact zone west/east, SAW/SAE = southern allopatric *E. euryale adyte* west/east.

We then inferred gene flow for each contact zone separately by running the program Admixture, assuming two genetic clusters, based on the PC analysis. Our results confirmed the phenotypic observations, i.e., that in even years primarily *isarica* individuals fly in both contact zones, whereas *adyte* was only found in the odd year (Figure 3A and C). Gene flow between the two lineages occurred but differed quantitatively between the contact zones. We found few unadmixed *adyte* but more early-stage hybrids in the western contact zone. This contrasts with the eastern contact zone, in which we primarily caught *adyte* in the odd year with only one single admixed individual. We estimated the level of genetic differentiation among sites and sampling years (Figure 4A and B). Allopatric *isarica* and *adyte* were strongly differentiated (west: *F*_ST_ = 0.576; east: *F*_ST_ = 0.656; Supplementary Table S3), while within-lineage differentiation among sites and/or years in *isarica* and *adyte* was low (all *F*_ST_ < 0.100). Consistent with the yearly phenotypic shifts, *F*_ST_ was high between even and odd years in the eastern contact zone, while the genetically admixed sample of 2019 from the western contact zone was genetically closer to *isarica*. The degree of genetic differentiation thus scales with the level of geographic isolation, with higher differentiation in the western than the eastern contact zone.

Because gene flow between the lineages may not be equal across the genome, e.g., as a result of adaptive introgression or the presence of barrier regions (Ravinet et al., 2017), we next tried to pinpoint such genomic regions. We first identified 135 single nucleotide polymorphisms (SNPs) (7.8% of all SNPs) that were fixed (i.e., *F*_ST_ = 1) between non-admixed allopatric *isarica* and *adyte*. Overall, there was more introgression at these loci in the western than the eastern contact zone as indicated by the higher number of early-stage hybrids and heterozygous sites (Figure 3B and D). Fitting genomic clines, we identified 34 outlier loci for the western and 15 for the eastern contact zone that showed less or more introgression than expected by chance. Of these, five loci overlapped between the two contact zones (Figure 3B and D; Supplementary Table S2). Out of the 44 outlier loci in total, 24 (54.5%) were within or nearby coding regions (Supplementary Table S2), and two of these belonged to the outliers that overlapped between both contact zones (apolipoprotein D, paired box protein *Pax-6*).

**Figure 5.**
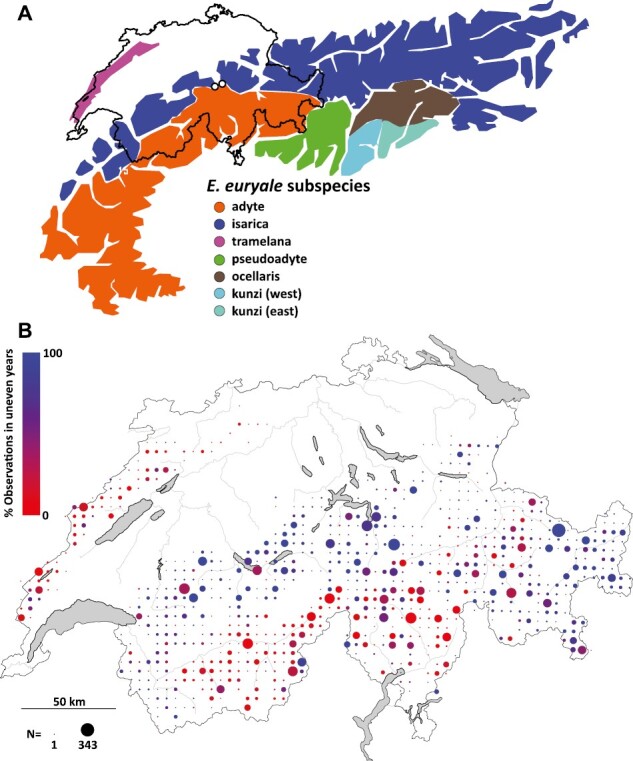
Distribution of *E. euryale* across the Alps and their phenology throughout Switzerland from 1876 to 2021. (A) Approximated distribution of *E. euryale* subspecies across the European Alps (modified from Johannesson et al., 2020). White circles indicate the location of the two studied contact zones. (B) Variation in phenology across Switzerland based on records from the Swiss faunistic database and individuals sampled for this study. Dot colors indicate the observed years of flight, ranging from purely flying in even (red) and odd (blue) years within 5 × 5 km grid cells. Dot size indicates sample size (*N*), ranging from 1 to 343.

Finally, we mapped the phenology of *E. euryale* across Switzerland by including more than 15,031 observations of the species, including the three subspecies *adyte*, *isarica*, and *tramelana* from the Swiss faunistic database (www.infofauna.ch). *E. euryale tramelana* does not occur in the Alps, but in the lower-elevated Jura mountains of western Switzerland (Figure 5). Phenology varied across 5 × 5 km grid cells, with individuals flying either predominantly in even years or in odd years within grid cells (Figure 5). The observational data did not distinguish between the three subspecies, but based on their suggested distributions (Figure 5; Cupedo & Doorenweerd, 2022; Sonderegger, 2005), phenological patterns seem to differ among the three subspecies. In the southern Alps where *adyte* occurs predominantly, records were mostly from odd years. In the northern Alps where *isarica* is common, records were primarily from even years. Also, *E. euralye tramelana* were caught predominantly in even years (Cupedo & Doorenweerd, 2022). Consistent with former suggestions that life cycles may not always be biennial (Kleckova et al., 2015; Sonderegger, 2005), some exceptions to the aforementioned patterns occurred across the respective distributions of all three lineages. It is also important to note that the observational data at hand reports the presence or absence rather than the abundance of *E. euryale*. Despite these caveats, our analysis highlights that allochronic differentiation seems to be a widespread phenomenon in *E. euryale*.

## Discussion

By studying two contact zones between a pair of closely related *Erebia* butterfly lineages, we identified phenotypic shifts between consecutive sampling years, with individuals caught in even or odd years resembling individuals that were collected at the allopatric sites of the respective lineage (Figure 1). Similar phenotypic shifts already occurred in these contact zones more than four decades ago (Figure 1C), suggesting that they have remained stable over time. The presence of stable and narrow contact zones has been reported also for other *Erebia* species (Augustijnen et al., 2022). The genomic analyses were consistent with the phenotypic shifts. Furthermore, they revealed differences in gene flow in the two contact zones associated with geographic isolation (Figure 3). We showed further that phenology in *E. euryale* varies at a broader geographic scale (Figure 5; Supplementary Figure S4). Together, results provide evidence for allochronic temporal isolation between the two *Erebia* lineages at their point of contact, with potential implications for their species integrity and speciation.

Allochrony, whereby temporal isolation causes reproductive isolation (Taylor & Friesen, 2017), is likely an important isolating mechanism at the zones of secondary contact in our system. From a theoretical perspective, allochrony has the potential to contribute to the process of species divergence under any geographic mode of speciation, ranging from sympatry to allopatry, and could also become the target for reinforcing selection following secondary contact by promoting isolation (Taylor & Friesen, 2017). Theory outlines three key components to demonstrate speciation by allochrony (Taylor & Friesen, 2017). The first is that the two lineages or species have to be sister species. The phylogeny of *Erebia* remains largely unresolved (Peña et al., 2015), which equally applies to the *E. euryale* complex (Cupedo & Doorenweerd, 2022). Both *isarica* and *adyte* are thought to be closely related and associated with distinct glacial refugia near the Alps. This contrasts, for example, with the subspecies *tramelana* from western Switzerland (Figure 5A), which is more closely related to *E. euryale* subspecies from the Pyrenees than the Alps (Cupedo & Doorenweerd, 2022). Consequently, *isarica* and *adyte* can be considered sister lineages.

Taylor and Friesen (2017) further highlight that differences in phenology require a genetic basis for allochronic speciation. In our system, allochrony is expressed by a biannual life cycle, which has been suggested for *E. euryale* from the Alps (Sonderegger, 2005; Wipking & Mengelkoch, 1994) and other parts of its range (Kleckova et al., 2015). However, the causes underlying biannual life cycles are unknown (Kleckova et al., 2015). Given the broadscale and often consistent pattern of a biennial phenology across Switzerland, including along different elevational gradients (Figure 5), it is likely that it has a genetic component, potentially with some plastic adjustments depending on environmental conditions. To disentangle the role of plasticity and shifts in allele frequencies, lab rearing experiments under natural conditions would be required. The intriguing aspect, however, is how it became rather synchronous within lineage. For *E. euryale* specifically, it has been hypothesized that biennial cycles could be driven by fluctuating parasitoid pressure, although empirical evidence is limited (Wipking & Mengelkoch, 1994). Other potential reasons include Allee effects, whereby densities for mating are too low if only a fraction of the population flies in one year. The annual occurrence of *isarica* individuals in the western contact zone could nevertheless be a result of cohort splitting, whereby some individuals would only take one year to develop (Crowley & Hopper, 2015). For allochrony to maintain narrow hybrid zones such as in our case, a further aspect is how the shift in periodicity between the lineages established. Again, given the broadscale pattern within lineages of *isarica* and *adyte*, it is likely that the shift happened prior to their range expansions as a random event.

The third requirement is that allochrony has to be the initial cause of divergence. Again, given the variation in phenology associated with the distinct *E. euryale* lineages (Figure 5; Cupedo & Doorenweerd, 2022), phenological differences are likely to be ancestral and thus have established in allopatry prior to secondary contact. Consequently, *E. euralye* is not a classic case of allochronic speciation (Taylor & Friesen, 2017), but an example where allochronic isolation contributes to maintain co-existence of both lineages at the contact zones for at least several decades (Figure 1). The alternative scenario whereby the contact zones represent recurrent recolonization events from allopatric sites seems unlikely as migration in *Erebia* is limited (Polic et al., 2014). While allochrony seems leaky to some degree (Figure 1; Supplementary Figure S4), our finding of overall consistent allochronic patterns between *isarica* and *adyte* as well as another *E. euryale* subspecies across Switzerland and about 40 generations back in time suggests that allochrony could be a rather stable isolation mechanism.

Differences in wing or color patterns as well as differences in genital morphology could similarly contribute to reproductive isolation. Indeed, differentiation in wing morphology or coloration contributes to prezygotic isolation in other butterflies through sexual selection (Lukhtanov et al., 2005; Merot et al., 2017). Likewise, differences in genital morphology have been suggested to result in prezygotic isolation through lock and key mechanisms among closely related butterflies, including between some *Erebia* species (Masly, 2012). We found that *E. euryale isarica* and *adyte* from the putative allopatric sites differed significantly from each other in wing pattern, i.e., the shape of the orange wing spots, and to a lesser extent in male genital morphology, but there was phenotypic overlap for both body parts (Figures 1 and 2). If strong divergent selection acted on these traits, we would predict increased phenotypic differentiation between *adyte* and *isarica* in the contact zones compared to their allopatric occurrences (Butlin & Smadja, 2018). Against this prediction, we found that individuals from the contact zones were phenotypically often similar to allopatric *adyte* or *isarica* in odd and even years, respectively, for both wing pattern and genital morphology (Figure 2). The phenotypic overlap of admixed with nonadmixed *adyte* or *isarica* individuals further suggests that wing pattern and genital morphology are unlikely to be under strong or even reinforcing selection or act as a strong prezygotic barrier.

Co-existence and widespread sympatry of sibling species is one of the possible outcomes of the speciation process (Coyne et al., 2004; Stankowski & Ravinet, 2021), but may not always be achieved. Instead, they may form zones of secondary contact, where novel barriers could evolve or existing barriers be reinforced to avoid maladaptive gene flow (Butlin & Smadja, 2018). The strengthening of reproductive isolation could, however, be swamped through recurrent maladaptive gene flow from populations outside the contact zone (Comeault & Matute, 2016). As a consequence, the evolutionary outcome of secondary contact, even between the same lineages, may differ among distinct contact zones if the selective regime and/or levels of gene flow vary. The latter is likely what we observed among our studied contact zones: gene flow between the two lineages was more pronounced in the western compared to the eastern contact zone (Figures 3 and 4). In the west, both *isarica* and *adyte* occurred in 2019, when in the eastern contact zone only a single early-stage hybrid was collected (Figure 3). Levels of population structure scale similarly between the two contact zones (Figure 4). Given the higher geographic connectivity of the western contact zone to putatively allopatric occurrences of either lineage, the observed pattern could also reflect higher levels of gene flow of allopatric *isarica* into the contact zone as well as to the allopatric *adyte* population. The increased phenotypic intergradation associated with such gene flow (Figure 1) could therefore be the result of introgression, promoting admixture, and prevent the build-up of additional prezygotic reproductive barriers that would counteract gene flow (Comeault & Matute, 2016). The two lineages are genomically as strongly divergent as other, taxonomically resolved, *Erebia* species that form secondary contact zones (Augustijnen et al., 2022; Lucek et al., 2020), but similarly fail to co-exist. Given that the potential for admixture at the contact zones depends on the degree of geographic isolation to more distant locations of either lineage could suggest that gene flow by individuals with weaker lineage-specific mate-recognition capabilities from outside the contact zones could swamp divergent selection in the contact zones (Servedio & Noor, 2003). The fine-scale patterns of gene flow seem to be dynamic (Figure 3), suggesting that barrier regions exist across the genome that are in some cases nearby to our genotyped regions (Butlin & Smadja, 2018; Seehausen et al., 2014).

Taken together, we show that our studied *E. euryale* lineages form very narrow contact zones that have been remarkably stable in their geographic positions over several decades (Figure 1; Rezbanyai-Reser, 1991). Gene flow between both lineages is still possible, but likely maladaptive given the limited level of admixture. The apparent stable co-existence of both lineages at a narrow scale seems to have been maintained by temporal isolation through allochrony. The lack of broadscale coexistence between the two lineages is consistent with other *Erebia* species (Augustijnen et al., 2022; Schmitt & Müller, 2007; Sonderegger, 2005) and *E. euryale* lineages (Cupedo, 2014) and may suggest other mechanisms such as niche conservatism (Klečková et al., 2022) or competitive exclusion through reproductive interference (Vodă et al., 2015). Our studied lineages therefore fall within the “grey zone” of late-stage speciation (Roux et al., 2016), where the level of genomic isolation is strong (Figures 3 and 4) but reproductive isolation incomplete, preventing large-scale co-existence in sympatry. Finally, allochrony could be a barrier to gene flow during secondary contact of lineages that diverged in allopatry in systems where gene flow is still possible.

## Methods

### Sample collection

Sampling focused on two contact zones of *E. euryale isarica* and *E. euryale adyte* in the central Alps of Switzerland (sites CZE and CZW in Figure 1A). In each year, we sampled between 9 and 71 specimens (Supplementary Table S1) along similar transects as outlined by Rezbanyai-Reser (1991), covering each of the described contact zones. We further sampled individuals from distant, putatively allopatric sites for *isarica* in the north (sites NAE and NAW) and *adyte* in the south (SAE and SAW). While allopatric sites were on different mountain ridges in the east, they were geographically connected in the west, although separated by less suitable open meadows used for agriculture. Individuals from both contact zones were collected in summers 2018–2021 during similar periods of the year (Supplementary Table S1). Allopatric individuals were collected in 2019. For each specimen, we recorded the GPS location where it was caught. Individuals were killed with acetone, their wings clipped for morphological investigation and the bodies stored at −20°C for morphological and genetic analyses.

We obtained pictures of the forewings from mounted individuals that were used for the original description of the contact zones (Rezbanyai-Reser, 1991) from the Natural History Museum Lucerne. These individuals were collected during summers 1978–1982 (Supplementary Table S4). Collection policy did not allow us to analyze genital morphology.

### Phenotypic data collection and analyses

Forewings of *Erebia* butterflies often differ phenotypically between species in terms of wing pattern, i.e., the size of the orange spots and the number, size, and position of eyespots (Cupedo, 2014; Sonderegger, 2005). To capture this phenotypic variation among our samples, we digitized the wings for all males from the contact zones and the respective distant sites (*N* = 425) with a flatbed scanner. We selected 27 geometric morphometric landmarks that covered wing pattern based on vein intersections or terminations as well as the extent and position of the white, black, and orange spots of the right dorsal forewing (Supplementary Figure S5; Supplementary Table S5). In case of damaged wings, we used the left forewing and flipped the picture horizontally. Landmarks were set in tpsDig2 v2.31 (Rohlf, 2010). We used MorphoJ v1.07a (Klingenberg, 2011) to calculate Procrustes coordinates. The same landmarks were used for the museum specimens for the right forewing.

Similar to wing pattern, male genital morphology is commonly used to distinguish between *E. euryale* subspecies (Cupedo, 2014; Sonderegger, 2005). We dissected the genital apparatus of each male and macerated it for 24 hr in 13% sodium hydroxide at room temperature. We stained the genitalia with a 3% eosin Y solution in 70% ethanol for 5 min and then washed the genitalia in 70% ethanol for 5 min, followed by another wash with 70% fresh ethanol for 20 min before storing the genitalia in 90% ethanol. We took pictures of the valves for a total of 402 specimens with a microscope (Leica M205 C, Leica Microsystems, Wetzlar, Germany) and digitized four linear measurements in ImageJ v1.52i (Schneider et al., 2012) based on five landmarks that covered the extent of the valve as well as the position of the first to third tooth (Supplementary Figure S6; Supplementary Table S1). For 23 males, genitalia were damaged and could not be measured. To account for size, we normalized the linear measures by the respective total length of the valve.

For wing pattern, including the shape of the orange spots, and genital morphology, we conducted separate PC analyses, based on the Procrustes coordinates or the linear measurements, respectively, in R v3.6.1 (R Core Team, 2021). Because hybrids may show phenotypes that are outside of either parental lineage, we used only specimen from the allopatric sites to construct the multivariate morphospace and then projected individuals from the contact zones into this morphospace following Lucek et al. (2014). To test for phenotypic changes in the contact zones between subsequent years, we employed ANOVAs on the scores of the PC axes that explained more than 5% of variation for wing pattern and genital morphology. The factors were the *biannual period* (2018/2019 and 2020/2021), *type of year* (even or odd), and *site* (contact zone), including the two two-way interactions with hybrid zone. Because of the uneven sampling of the contact zones over time, only the factors *type of year* and *site*, without interaction, was included in the analysis of historic samples.

### Genomic sample preparations and analyses

We extracted DNA from thorax tissue using the Qiagen DNA Blood and Tissue kit (Qiagen, Zug, Switzerland). Genotyping of the individuals was conducted by RAD with the restriction enzyme *Sbf*I for samples collected in 2018 and 2019 and the compatible enzyme *Pst*I for all 2020 and part of the 2019 samples. RAD library preparation and sequencing was outsourced to Floragenex, Inc. (Portland, OR, USA). Libraries were single-end sequenced in three batches on an Illumina HiSeq2500. Only a subset of the collected individuals could be genotyped (*N* = 180), aiming to cover the observed phenotypic diversity for both wing pattern and genital morphology.

We filtered the obtained reads to have intact restriction sites, and demultiplexed and barcode-trimmed them with the function process_radtags from Stacks v1.48 (Catchen et al., 2013). Using the FastX toolkit (http://hannonlab.cshl.edu/fastx_toolkit/), we removed reads containing bases with a Phred quality score <10 or more than 5% of base pairs with quality <30. We then mapped the reads of each individual against the reference genome of *Erebia ligea* (Lohse et al., 2022), a close relative to *E. euryale*. We used MiniMap2 (Li, 2018) to map and SAMTools v1.7.20 (Li et al., 2009) to filter reads with a mapping quality < 20. We called variants with BCFTools (Li, 2011) and extracted SNP sites covered by the *Sbf*I dataset and obtained the same sites from the *Pst*I dataset. Using VCFtools v0.1.14 (Danecek et al., 2011) we filtered the merged VCF file by removing genotypes with quality < 20, a depth < 6, sites with > 25% missing data, nonbiallelic sites, indels, and SNPs with minor allele frequency < 0.03. We removed 15 individuals due to high rates of missing sites. Our filtering resulted in 1,727 SNPs for 180 individuals.

In a first step, genomic data were used to confirm shifts in *adyte* and *isarica* abundances in the contact zones and to estimate the genetic structure across sites and years. Using GenoDive v3.04 (Meirmans, 2020), we performed PC analyses on the genomic data separately for the western and eastern contact zone, each including individuals from the respective allopatric sites. Because the leading PCs separated *adyte* and *isarica*, we further inferred the potential for interspecific gene flow between *adyte* and *isarica* with Admixture v1.3.0 (Alexander et al., 2009). Admixture implements a maximum likelihood approach to estimate ancestry, and we ran the program for all individuals assuming two genetic clusters (*K* = 2). Furthermore, we estimated the level of genetic differentiation (*F*_ST_) with GenoDive for each locus between *isarica* and *adyte* individuals from the distant locations that showed no gene flow in the Admixture analysis.

In a second step, we quantified introgression. We identified the SNPs that were fixed (*F*_ST_ = 1) for different alleles between the subspecies in each of the two contact zones and then extracted the allele frequencies of these SNPs for all individuals. With the R package introgress (Gompert & Buerkle, 2010), we fitted genomic clines for each locus for all individuals from the western and the eastern contact zone, respectively. Specifically, introgress compares each genomic cline against a null model of neutral introgression to identify SNPs that show patterns of introgression inconsistent with neutral expectations. These non-neutral SNPs could reflect reduced introgression or a deficit of heterozygotes, possibly linked to genes that contribute to reproductive isolation; alternatively, outliers may indicate increased introgression or an excess of heterozygotes due to hybrid vigor (Gompert & Buerkle, 2010). Deviation from neutral expectation was assessed with 1,000 permutation steps for each SNP position, applying a false discovery rate correction across all significant values for each contact zone. Because the *E. ligea* genome lacks an annotation, we extracted 1,000 bps around each outlier locus and used blastx (Boratyn et al., 2013) to assess if outliers overlapped with coding regions. We employed blastx for each sequence against the protein database of *Maniola jurtina*, *Pararge aegeria*, and *Bombyx mori*.

### Phenology of *E. euryale* across Switzerland

Finally, to assess the phenology of *E. euryale* spp. in a broader context, we obtained a total of 15,031 observation records in addition to our 425 individuals from the Swiss faunistic database info fauna (www.infofauna.ch). Records stem from 1876 to 2021 with a large majority of individuals being reported since 1970 (Supplementary Figure S7). We projected a 5 × 5 km grid over Switzerland and calculated for each cell the percentage of observations that occurred in odd years. We did this using either all available data or for records of the faunistic data base between 1960 and 1979, 1980 and 1999, and 2000 and 2019.

## Supplementary Material

qrad046_suppl_Supplementary_Figures

qrad046_suppl_Supplementary_Tables

## Data Availability

All genomic data are deposited on NCBI (BioProject ID: PRJNA1019795), phenotypic data and codes are available from Zenodo (DOI: 10.5281/zenodo.8369364).

## References

[CIT0001] Alexander, D. H., Novembre, J., & Lange, K. (2009). Fast model-based estimation of ancestry in unrelated individuals. Genome Research, 19(9), 1655–1664. 10.1101/gr.094052.109 19648217 PMC2752134

[CIT0002] Augustijnen, H., Patsiou, T., & Lucek, K. (2022). Secondary contact rather than coexistence—*Erebia* butterflies in the Alps. Evolution, 76, 2669–2686. 36117267 10.1111/evo.14615 PMC9828779

[CIT0003] Boratyn, G. M., Camacho, C., Cooper, P. S., Coulouris, G., Fong, A., Ma, N., Madden, T. L, Matten, W. T., McGinnis, S. D., Merezhuk, Y., Raytselis, Y., Sayers, E. W., Tao, T., Ye, J., & Zaretskaya, I. (2013). BLAST: A more efficient report with usability improvements. Nucleic Acids Research, 41(Web Server issue), W29–W33. 10.1093/nar/gkt282 23609542 PMC3692093

[CIT0004] Butlin, R. K., & Smadja, C. M. (2018). Coupling, reinforcement, and speciation. The American Naturalist, 191(2), 155–172. 10.1086/695136 29351021

[CIT0005] Catchen, J., Hohenlohe, P. A., Bassham, S., Amores, A., & Cresko, W. A. (2013). Stacks: An analysis tool set for population genomics. Molecular Ecology, 22(11), 3124–3140. 10.1111/mec.12354 23701397 PMC3936987

[CIT0006] Comeault, A. A., & Matute, D. R. (2016). Reinforcement’s incidental effects on reproductive isolation between conspecifics. Current Zoology, 62(2), 135–143. 10.1093/cz/zow002 29491901 PMC5804225

[CIT0007] Coyne, J. A., Coyne, H. A. & Orr, H.A. (2004). Speciation. Oxford University Press.

[CIT0008] Crowley, P. H., & Hopper, K. R. (2015). Mechanisms for adaptive cohort splitting. Ecological Modelling, 308, 1–13. 10.1016/j.ecolmodel.2015.03.018

[CIT0009] Cupedo, F. (2010). A revision of the infraspeciﬁc structure of *Erebia euryale* (Esper, 1805) (Nymphalidae: Satyrinae). Nota Lepidopterologica, 33, 85–106.

[CIT0010] Cupedo, F. (2014). Reproductive isolation and intraspecific structure in Alpine populations of *Erebia euryale* (Esper, 1805) (Lepidoptera, Nymphalidae, Satyrinae). Nota Lepidopterologica, 37(1), 19–36. 10.3897/nl.37.7960

[CIT0011] Cupedo, F., & Doorenweerd, C. (2022). Mitochondrial DNA-based phylogeography of the large ringlet *Erebia euryale* (Esper, 1805) suggests recurrent Alpine-Carpathian disjunctions during Pleistocene (Nymphalidae, Satyrinae). Nota Lepidopterologica, 45, 65–86. 10.3897/nl.45.68138

[CIT0012] Danecek, P., Auton, A., Abecasis, G., Albers, C. A., Banks, E., DePristo, M. A., Handsaker, R. E., Lunter, G., Marth, G. T., Sherry, S. T., McVean, G., & Durbin, R.; 1000 Genomes Project Analysis Group (2011). The variant call format and VCFtools. Bioinformatics, 27(15), 2156–2158. 10.1093/bioinformatics/btr330 21653522 PMC3137218

[CIT0013] Gompert, Z., & Buerkle, C. (2010). introgress: A software package for mapping components of isolation in hybrids. Molecular Ecology Resources, 10, 378–384. 21565033 10.1111/j.1755-0998.2009.02733.x

[CIT0014] Green, C. -P., Ratcliffe, N., Mattern, T., Thompson, D., Lea, M. -A., Wotherspoon, S., Borboroglu, P. G., Ellenberg, U., Morrison, K. W., Pütz, K., Sagar, P. M., Seddon, P. J., Torres, L. G., & Hindell, M. A. (2022). The role of allochrony in influencing interspecific differences in foraging distribution during the non-breeding season between two congeneric crested penguin species. PLoS One, 17(2), e0262901. 10.1371/journal.pone.0262901 35139102 PMC8827451

[CIT0015] Hudson, A. G., Vonlanthen, P., & Seehausen, O. (2011). Rapid parallel adaptive radiations from a single hybridogenic ancestral population. Proceedings of the Royal Society B: Biological Sciences, 278(1702), 58–66. 10.1098/rspb.2010.0925 PMC2992718 20685705

[CIT0016] Inskeep, K. A., Doellman, M. M., Powell, T. H. Q., Berlocher, S. H., Seifert, N. R., Hood, G. R., Ragland, G. J., Meyers, P. J., & Feder, J. L. (2022). Divergent diapause life history timing drives both allochronic speciation and reticulate hybridization in an adaptive radiation of *Rhagoletis* flies. Molecular Ecology, 31(15), 4031–4049. 10.1111/mec.15908 33786930

[CIT0018] Johannesson, K., Le Moan, A., Perini, S., & André, C. (2020). A Darwinian laboratory of multiple contact zones. Trends in Ecology & Evolution, 35(11), 1021–1036. 10.1016/j.tree.2020.07.015 32912631

[CIT0056] Klečková, I., Klečka, J., Fric, Z.F., Česánek, M., Dutoit, L., Pellissier, L., & Matos-Maraví, P. (2022). Climatic niche conservatism and ecological diversification in the holarctic cold-dwelling butterfly genus *Erebia*. Insect Systematics and Diversity, 7, 2. 10.1093/isd/ixad002

[CIT0020] Kleckova, I., Vrba, P., & Konvicka, M. (2015). Quantitative evidence for spatial variation in the biennial life cycle of the mountain butterfly *Erebia euryale* (Lepidoptera: Nymphalidae) in the Czech Republic. European Journal of Entomology, 112(1), 114–119. 10.14411/eje.2015.003

[CIT0021] Klingenberg, C. P. (2011). MorphoJ: An integrated software package for geometric morphometrics. Molecular Ecology Resources, 11(2), 353–357. 10.1111/j.1755-0998.2010.02924.x 21429143

[CIT0022] Kulmuni, J., Butlin, R. K., Lucek, K., Savolainen, V., & Westram, A. M. (2020). Towards the completion of speciation: The evolution of reproductive isolation beyond the first barriers. Philosophical Transactions of the Royal Society of London, Series B: Biological Sciences, 375(1806), 20190528. 10.1098/rstb.2019.0528 32654637 PMC7423269

[CIT0023] Li, H. (2011). A statistical framework for SNP calling, mutation discovery, association mapping and population genetical parameter estimation from sequencing data. Bioinformatics, 27(21), 2987–2993. 10.1093/bioinformatics/btr509 21903627 PMC3198575

[CIT0024] Li, H. (2018). Minimap2: Pairwise alignment for nucleotide sequences. Bioinformatics, 34(18), 3094–3100. 10.1093/bioinformatics/bty191 29750242 PMC6137996

[CIT0025] Li, H., Handsaker, B., Wysoker, A., Fennell, T., Ruan, J., Homer, N., Marth, G., Abecasis, G., & Durbin, R.; 1000 Genome Project Data Processing Subgroup (2009). The Sequence Alignment/Map format and SAMtools. Bioinformatics, 25(16), 2078–2079. 10.1093/bioinformatics/btp352 19505943 PMC2723002

[CIT0026] Lohse, K., Hayward, A., Laetsch, D. R., Vila, R., & Lucek, K.; Wellcome Sanger Institute Tree of Life programme (2022). The genome sequence of the Arran brown, *Erebia ligea* (Linnaeus, 1758). Wellcome Open Research, 7, 259. 10.12688/wellcomeopenres.18115.1 37346774 PMC10280028

[CIT0027] Lucek, K., Bouaouina, S., Jospin, A., Grill, A., & de Vos, J. M. (2021). Prevalence and relationship of endosymbiotic Wolbachia in the butterfly genus Erebia. BMC Ecology and Evolution, 21(1), 95. 10.1186/s12862-021-01822-9 34020585 PMC8140509

[CIT0028] Lucek, K., Butlin, R. K., & Patsiou, T. (2020). Secondary contact zones of closely‐related *Erebia* butterflies overlap with narrow phenotypic and parasitic clines. Journal of Evolutionary Biology, 33(9), 1152–1163. 10.1111/jeb.13669 32573833

[CIT0029] Lucek, K., Sivasundar, A., & Seehausen, O. (2014). Disentangling the role of phenotypic plasticity and genetic divergence in contemporary ecotype formation during a biological invasion. Evolution, 68(9), 2619–2632. 10.1111/evo.12443 24766190

[CIT0030] Lukhtanov, V. A., Kandul, N. P., Plotkin, J. B., Dantchenko, A. V., Haig, D., & Pierce, N. E. (2005). Reinforcement of pre-zygotic isolation and karyotype evolution in *Agrodiaetus* butterflies. Nature, 436(7049), 385–389. 10.1038/nature03704 16034417

[CIT0031] Masly, J. P. (2012). 170 Years of “lock-and-key”: Genital morphology and reproductive isolation. International Journal of Evolutionary Biology, 2012, 1–10. 10.1155/2012/247352 PMC3235471 22263116

[CIT0032] Meirmans, P. G. (2020). Genodive version 3.0: Easy‐to‐use software for the analysis of genetic data of diploids and polyploids. Molecular Ecology Resources, 20(4), 1126–1131. 10.1111/1755-0998.13145 32061017 PMC7496249

[CIT0033] Merot, C., Salazar, C., Merrill, R. M., Jiggins, C. D., & Joron, M. (2017). What shapes the continuum of reproductive isolation? Lessons from *Heliconius* butterflies. Proceedings of the Royal Society B: Biological Sciences, 284(1856), 20170335. 10.1098/rspb.2017.0335 PMC5474069 28592669

[CIT0034] Peña, C., Witthauer, H., Klec, I., Fric, K., & Wahlberg, N. (2015). Adaptive radiations in butterflies: Evolutionary history of the genus Erebia (Nymphalidae: Satyrinae). Biological Journal of the Linnean Society, 116, 449–467.

[CIT0035] Polic, D., Fiedler, K., Nell, C., & Grill, A. (2014). Mobility of ringlet butterflies in high-elevation alpine grassland: Effects of habitat barriers, resources and age. Journal of Insect Conservation, 18(6), 1153–1161. 10.1007/s10841-014-9726-5

[CIT0036] R Core Team. (2021). R: A language and environment for statistical computing. R Foundation for Statistical Computing. https://www.R-project.org/.

[CIT0037] Ravinet, M., Faria, R., Butlin, R. K., Galindo, J., Bierne, N., Rafajlović, M., Noor, M. A. F., Mehlig, B., & Westram, A. M. (2017). Interpreting the genomic landscape of speciation: A road map for finding barriers to gene flow. Journal of Evolutionary Biology, 30(8), 1450–1477. 10.1111/jeb.13047 28786193

[CIT0038] Rezbanyai-Reser, L. (1991). Die drei Zentralschweizer Kontaktstellen der *Erebia euryale* Unterarten *isarica* Heyne und *adyte* Hübner. Entomologische Berichte Luzern, 25, 77–90.

[CIT0039] Rohlf, F.J. (2010). tpsDig v2. 16. Free software available. Available on: https://www.sbmorphometrics.org/soft-dataacq.html (accessed June 22, 2021).

[CIT0040] Rosser, N., Seixas, F., & Mallet, J. (2022). Sympatric speciation by allochrony? Molecular Ecology, 31(15), 3975–3978. 10.1111/mec.16599 35789010

[CIT0041] Roux, C., Fraïsse, C., Romiguier, J., Anciaux, Y., Galtier, N., & Bierne, N. (2016). Shedding light on the grey zone of speciation along a continuum of genomic divergence. PLoS Biology, 14(12), e2000234. 10.1371/journal.pbio.2000234 28027292 PMC5189939

[CIT0042] Schmitt, T., & Haubrich, K. (2008). The genetic structure of the mountain forest butterfly *Erebia euryale* unravels the late Pleistocene and postglacial history of the mountain coniferous forest biome in Europe. Molecular Ecology, 17(9), 2194–2207. 10.1111/j.1365-294X.2007.03687.x 18266631

[CIT0043] Schmitt, T., & Müller, P. (2007). Limited hybridization along a large contact zone between two genetic lineages of the butterfly *Erebia medusa* (Satyrinae, Lepidoptera) in Central Europe. Journal of Zoological Systematics and Evolutionary Research, 45(1), 39–46. 10.1111/j.1439-0469.2006.00404.x

[CIT0044] Schneider, C. A., Rasband, W. S., & Eliceiri, K. W. (2012). NIH Image to ImageJ: 25 Years of image analysis. Nature Methods, 9(7), 671–675. 10.1038/nmeth.2089 22930834 PMC5554542

[CIT0045] Seehausen, O., Butlin, R. K., Keller, I., Wagner, C. E., Boughman, J. W., Hohenlohe, P. A., Peichel, C. L, Saetre, G. -P., Bank, C., Brännström, A., Brelsford, A., Clarkson, C. S., Eroukhmanoff, F., Feder, J. L, Fischer, M. C, Foote, A. D., Franchini, P., Jiggins, C. D., Jones, F. C, … Widmer, A. (2014). Genomics and the origin of species. Nature Reviews Genetics, 15(3), 176–192. 10.1038/nrg3644 24535286

[CIT0046] Servedio, M. R., & Noor, M. A. F. (2003). The role of reinforcement in speciation: Theory and data. Annual Review in Ecology, Evolution and Systematics, 34, 339–364.

[CIT0047] Simon, C., Tang, J., Dalwadi, S., Staley, G., Deniega, J., & Unnasch, T. R. (2000). Genetic evidence for assortative mating between 13-Year cicadas and sympatric “17-year Cicadas with 13-year life cycles” provides support for allochronic speciation. Evolution, 54(4), 1326–1336. 10.1111/j.0014-3820.2000.tb00565.x 11005299

[CIT0048] Sonderegger, P. (2005). *Die Erebien der Schweiz.* P. Sonderegger, Brügg bei Biel.

[CIT0049] Stankowski, S., & Ravinet, M. (2021). Defining the speciation continuum. *Evolution*, 75(6), 1256–1273. 10.1111/evo.14215 33754340

[CIT0050] Tang, Q., Burri, R., Liu, Y., Suh, A., Sundev, G., Heckel, G., & Schweizer, M. (2022). Seasonal migration patterns and the maintenance of evolutionary diversity in a cryptic bird radiation. Molecular Ecology, 31(2), 632–645. 10.1111/mec.16241 34674334 PMC9298432

[CIT0051] Taylor, R. S., & Friesen, V. L. (2017). The role of allochrony in speciation. Molecular Ecology, 26(13), 3330–3342. 10.1111/mec.14126 28370658

[CIT0057] Vodă, R., Dapporto, L., Dincă, V., & Vila, R. (2015). Why do cryptic species tend not to co-occur? A case study on two cryptic pairs of butterflies. PLoS One, 10, e0117802. 10.1371/journal.pone.0117802 25692577 PMC4334660

[CIT0054] Weber, M. G., & Strauss, S. Y. (2016). Coexistence in close relatives: Beyond competition and reproductive isolation in sister taxa. Annual Review of Ecology, Evolution, and Systematics, 47(1), 359–381. 10.1146/annurev-ecolsys-112414-054048

[CIT0055] Wipking, W. & Mengelkoch, C. (1994). Control of alternate-year flight activities in high-alpine Ringlet butterflies (*Erebia, Satyridae*) and Burnet moths (*Zygaena*, Zygaenidae) from temperate environments. In H. V. Danks (Ed.), Insect life-cycle polymorphism (pp. 313–347). Springer.

