# Supplementary material for: Asynchronous life cycles contribute to reproductive isolation between two Alpine butterflies
Source: Evol Lett. 2023 Oct 7;7(6):436–46. doi: 10.1093/evlett/qrad046 (PMC10692998; doi:10.1093/evlett/qrad046)
Supplement: qrad046_suppl_Supplementary_Figures [file qrad046_suppl_supplementary_figures.pdf]

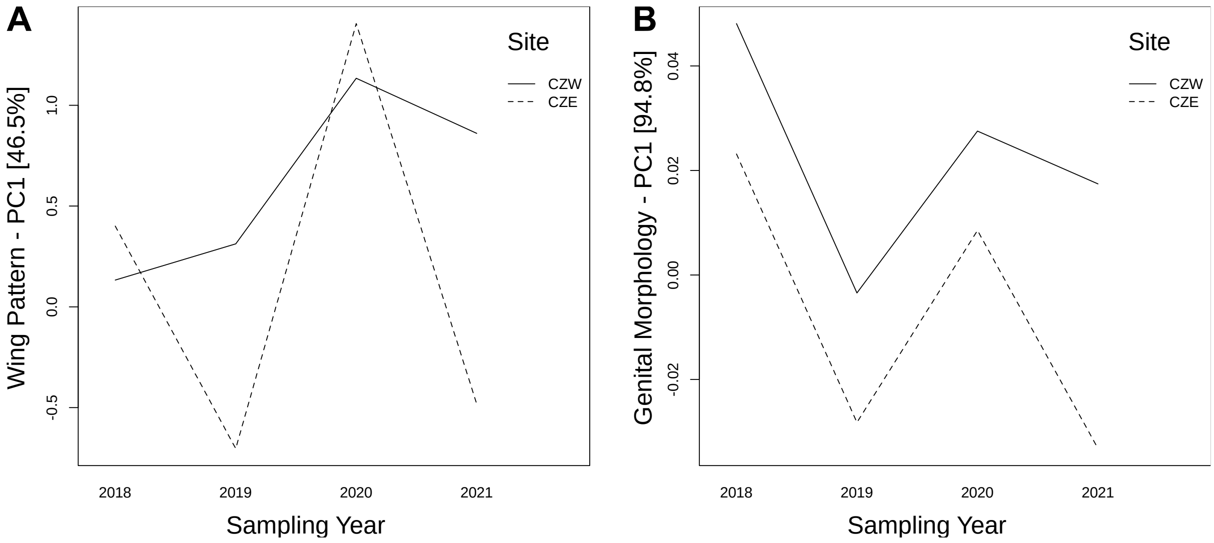

Fig. S1: Interaction plots showing the interaction between sampling year and contact zone as collection site for the leading PC axis for wing shape (A) and genital morphology (B).

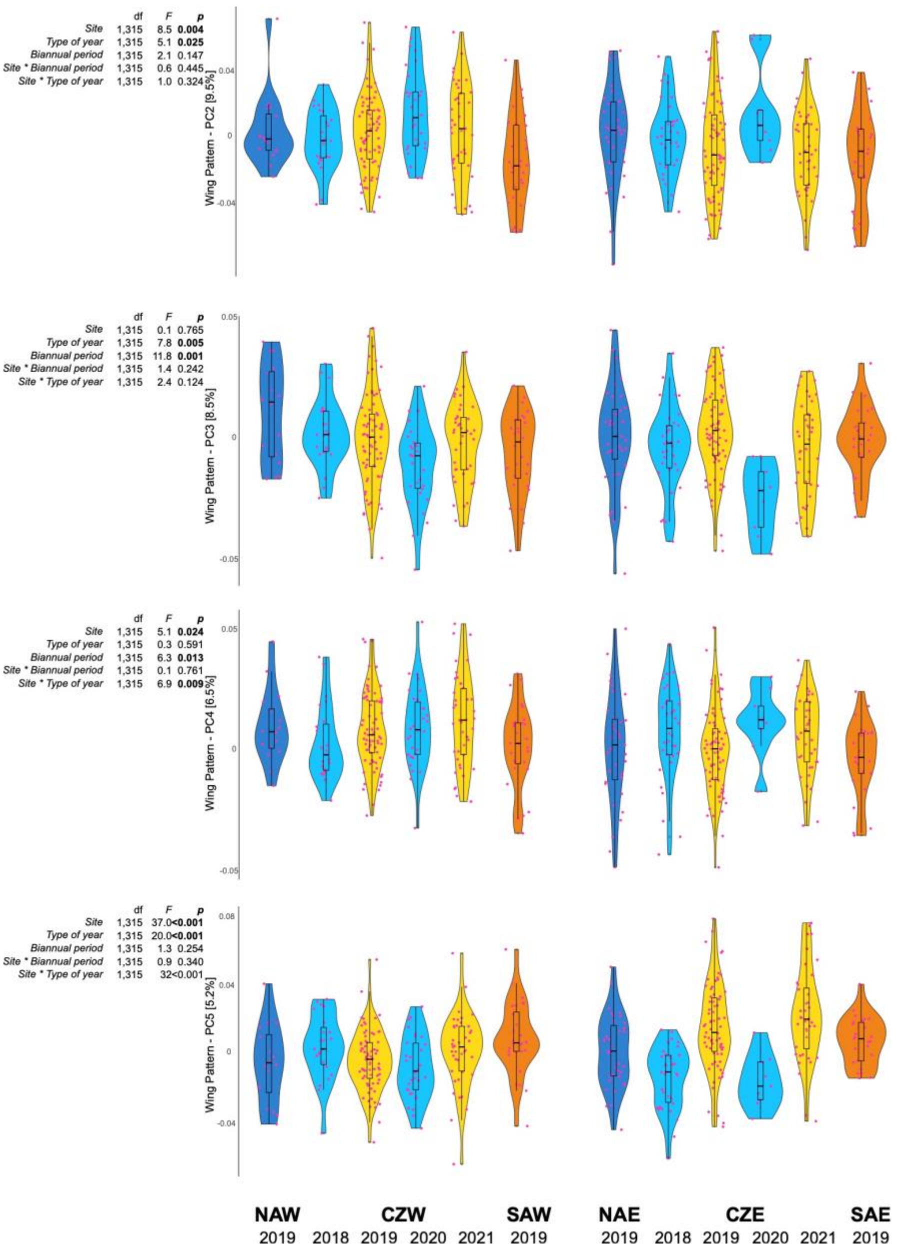

Fig. S2: Violin plots for the principal component (PC) axes 2-5 for wing shape, separately for sampling site and year with the respective ANOVA model. NAW/NAE= northern allopatric *E. euryale isarica* west/east; CZW/CZE= contact zone west/east; SAW/SAE= southern allopatric *E. euryale adyte* west/east. Dark colours indicate allopatric populations (blue = *isarica*, orange = *adyte*), light colours alternating years of sampling at the contact zones. For each PC, the respective ANOVA statistics are indicated, using *contact zone*, *type of year* (even or odd) and *biannual period* (2018/2019 and 2020/2021) as factors.

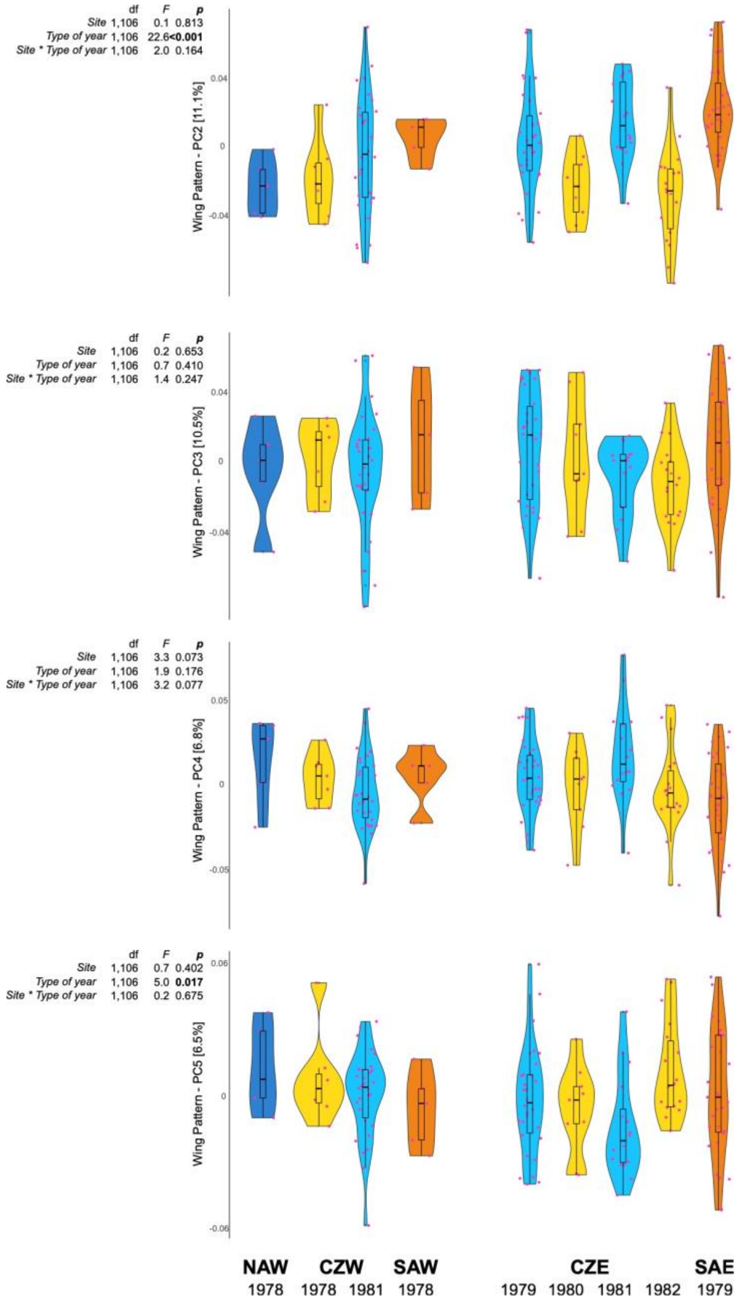

Fig. S3: Violin plots for the principal component (PC) axes 2-5 for wing shape of historic samples, separately for sampling site and year with the respective ANOVA model. NAW/NAE= northern allopatric *E. euryale isarica* west/east; CZW/CZE= contact zone west/east; SAW/SAE= southern allopatric *E. euryale adyte* west/east. Dots indicate individual PC scores. Dark colours indicate allopatric populations (blue = *isarica*, orange = *adyte*), light colours alternating years of sampling at the contact zones. For each PC, the respective ANOVA statistics are indicated, using *contact zone* and *type of year* (even or odd) as factors.

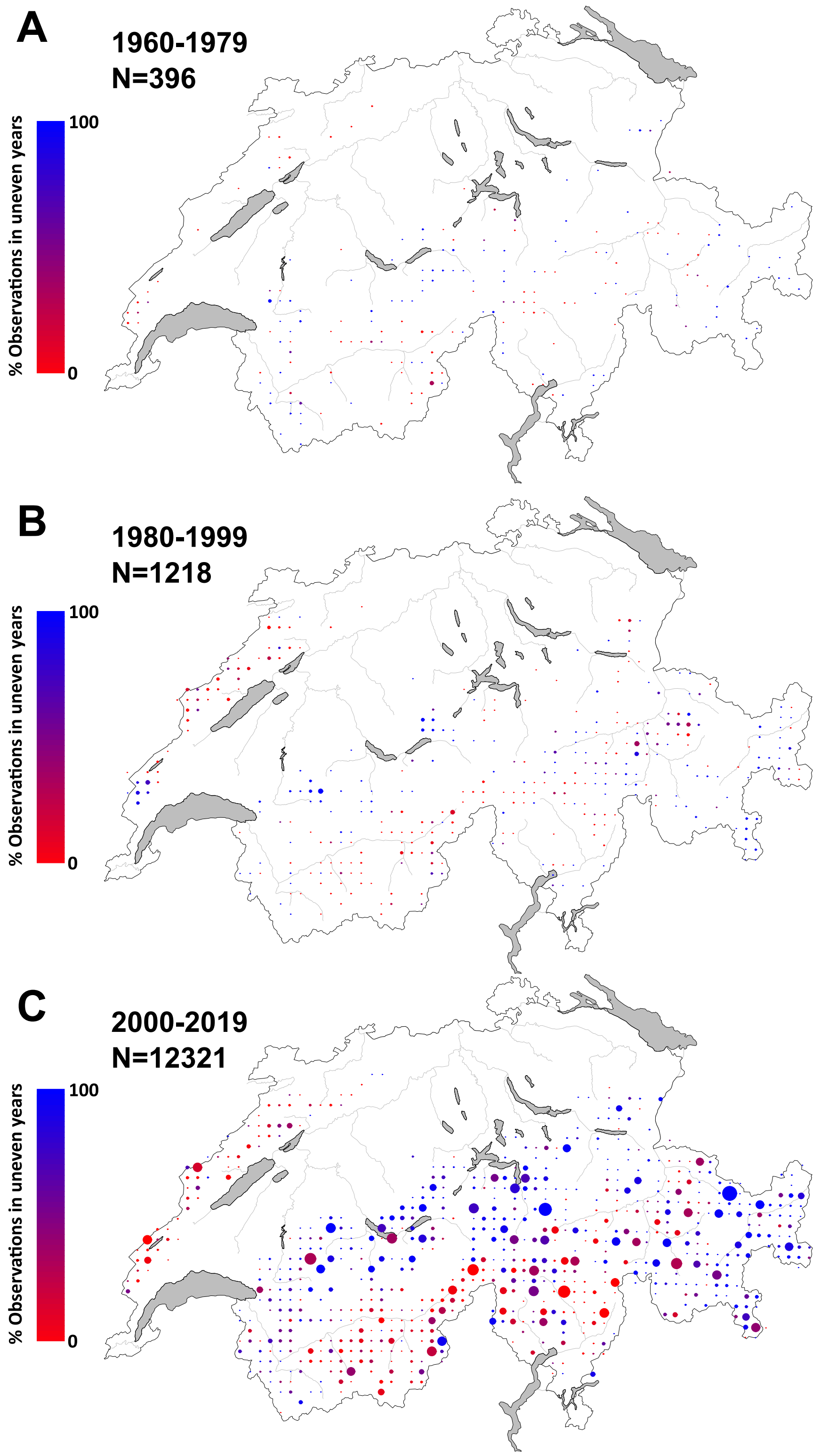

Fig. S4: Variation in phenology across Switzerland based on records from the Swiss faunistic database during three time periods A) 1960-79, B) 1980-99, C) 2000-2019. Dot colours indicate the observed years of flight, ranging from purely flying in even (red) and odd (blue) years within 5x5 km grid cells. Dot size are scaled to sample sizes (N).

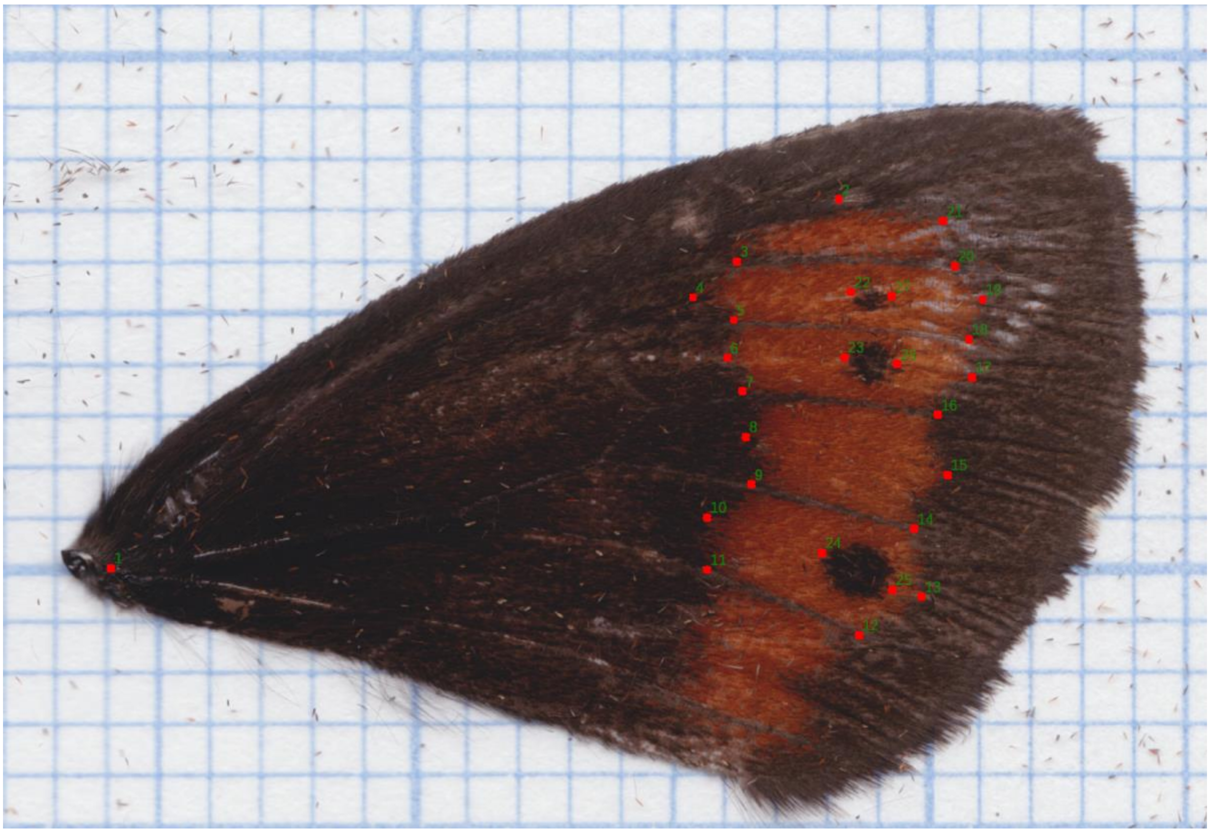

Fig. S5: Upper forewing showing the landmarks used for the geometric morphometric analysis.

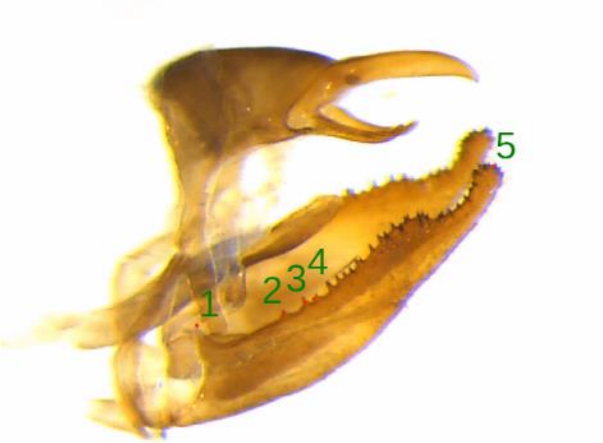

Fig S6: Male valve with the five digitized landmarks related to the valve base (1), the first, second and third tooth (2-4) and the valve tip (5). Based on these landmarks we estimated the distances of the first, second and third tooth from the distal end, the distance between the first and second and the second and third tooth. All measures were divided by the valve length.

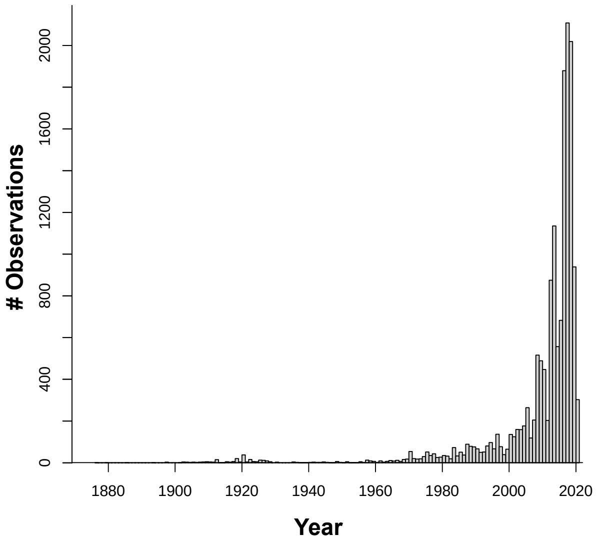

Fig. S7: Temporal distribution of all available records of the Swiss faunistic database between 1876 and 2021.
